# Supplementary material for: Exploring the Antioxidant Properties of Caffeoylquinic and Feruloylquinic Acids: A Computational Study on Hydroperoxyl Radical Scavenging and Xanthine Oxidase Inhibition
Source: Antioxidants (Basel). 2023 Aug 25;12(9):1669. doi: 10.3390/antiox12091669 (PMC10526077; doi:10.3390/antiox12091669)
Supplement: Supplementary file 1 [file antioxidants-12-01669-s001.zip › antioxidants-2525317-supplementary.pdf]

SUPPORTING INFORMATION

Exploring the Antioxidant Properties of Caffeoylquinic and Feruloylquinic Acids: A Computational Study on Hydroperoxyl Radical Scavenging and Xanthine Oxidase Inhibition

Houssem Boulebd[1,*], Miguel Carmena-Bargueño[2], Horacio Pérez-Sánchez[2]

[1] Department of Chemistry, Faculty of Exact Science, University of Constantine 1, Constantine 25000, Algeria

[2] Structural Bioinformatics and High-Performance Computing Research Group (BIO-HPC), Computer Engineering Department, Universidad Católica de Murcia (UCAM), Campus de los Jerónimos 135, 30107 Guadalupe, Spain; mcarmena@ucam.edu (M.C.-B.); hperez@ucam.edu (H.P.-S.)

* Correspondence: boulebd.houssem@umc.edu.dz

Table S1. The cartesian coordinates and energies of the TSs of the reaction between 5-CQA and 5-FQA and HOO• calculated at M06-2X/6-311++G(d,p) level in physiological media.

<table>
<tr><td colspan="5">5CQA W RAF C8 dianion TS</td></tr>
<tr><td colspan="4">Cartesian Coordinates</td><td>Frequency and Energy</td></tr>
<tr><td>C</td><td>-3.53034900</td><td>-0.17879900</td><td>-0.18519900</td><td rowspan="40">Zero-point Energy Correction = 0.328347 Hartree
Thermal Correction to Energy = 0.354144 Hartree
Thermal Correction to Enthalpy = 0.355088 Hartree
Thermal Correction to Free Energy = 0.270157 Hartree
EE + Zero-point Energy = -1447.177 Hartree
EE + Thermal Energy Correction = -1447.1512 Hartree
EE + Thermal Enthalpy Correction = -1447.1503 Hartree
EE + Thermal Free Energy Correction = -1447.2352 Hartree</td></tr>
<tr><td>C</td><td>-4.06455300</td><td>0.90339300</td><td>-0.94672300</td></tr>
<tr><td>C</td><td>-5.40184500</td><td>1.15778500</td><td>-0.94734600</td></tr>
<tr><td>C</td><td>-6.33897600</td><td>0.35643200</td><td>-0.18304300</td></tr>
<tr><td>C</td><td>-5.78087300</td><td>-0.72033900</td><td>0.58105400</td></tr>
<tr><td>C</td><td>-4.43724100</td><td>-0.97349800</td><td>0.57862100</td></tr>
<tr><td>C</td><td>-2.13572400</td><td>-0.40509400</td><td>-0.19561700</td></tr>
<tr><td>C</td><td>-1.48416800</td><td>-1.40939300</td><td>0.49152200</td></tr>
<tr><td>C</td><td>-0.03334000</td><td>-1.60645800</td><td>0.40012900</td></tr>
<tr><td>O</td><td>0.62296000</td><td>-0.54590400</td><td>-0.08951000</td></tr>
<tr><td>O</td><td>0.52796500</td><td>-2.62703500</td><td>0.76372100</td></tr>
<tr><td>C</td><td>2.06598100</td><td>-0.56423300</td><td>-0.07471400</td></tr>
<tr><td>C</td><td>2.52411600</td><td>0.87101900</td><td>0.11930900</td></tr>
<tr><td>C</td><td>4.05592000</td><td>0.94442000</td><td>0.16600300</td></tr>
<tr><td>C</td><td>4.66178800</td><td>0.29434000</td><td>-1.08577700</td></tr>
<tr><td>C</td><td>4.12513600</td><td>-1.09712500</td><td>-1.39457700</td></tr>
<tr><td>C</td><td>2.59351800</td><td>-1.11534700</td><td>-1.39144800</td></tr>
<tr><td>O</td><td>2.11165000</td><td>-2.42579500</td><td>-1.66519700</td></tr>
<tr><td>O</td><td>4.52151900</td><td>0.28857100</td><td>1.34178300</td></tr>
<tr><td>O</td><td>-7.57022400</td><td>0.64163800</td><td>-0.22535300</td></tr>
<tr><td>O</td><td>-5.93267400</td><td>2.18651000</td><td>-1.67117800</td></tr>
<tr><td>O</td><td>4.63652300</td><td>-2.03409500</td><td>-0.44795800</td></tr>
<tr><td>C</td><td>4.48481300</td><td>2.43606600</td><td>0.22931100</td></tr>
<tr><td>O</td><td>4.18037800</td><td>3.17050300</td><td>-0.73581900</td></tr>
<tr><td>O</td><td>5.10611200</td><td>2.78456400</td><td>1.26559700</td></tr>
<tr><td>H</td><td>-3.39689300</td><td>1.52694500</td><td>-1.53270400</td></tr>
<tr><td>H</td><td>-6.46313400</td><td>-1.32544200</td><td>1.16783400</td></tr>
<tr><td>H</td><td>-4.05382600</td><td>-1.79157500</td><td>1.17737200</td></tr>
<tr><td>H</td><td>-1.53256400</td><td>0.31541900</td><td>-0.73924600</td></tr>
<tr><td>H</td><td>2.39451700</td><td>-1.19120500</td><td>0.75652100</td></tr>
<tr><td>H</td><td>2.15583600</td><td>1.47816700</td><td>-0.71201800</td></tr>
<tr><td>H</td><td>2.11190400</td><td>1.26617100</td><td>1.05124800</td></tr>
<tr><td>H</td><td>4.42715000</td><td>0.93005900</td><td>-1.94291000</td></tr>
<tr><td>H</td><td>5.74974300</td><td>0.25411100</td><td>-0.98604000</td></tr>
<tr><td>H</td><td>4.45710500</td><td>-1.38972200</td><td>-2.39662700</td></tr>
<tr><td>H</td><td>2.23995800</td><td>-0.47452400</td><td>-2.20578000</td></tr>
<tr><td>H</td><td>1.96895100</td><td>-2.88894600</td><td>-0.82843900</td></tr>
<tr><td>H</td><td>4.93150400</td><td>0.99838200</td><td>1.86491300</td></tr>
<tr><td>H</td><td>4.33136000</td><td>-2.91112300</td><td>-0.71057700</td></tr>
<tr><td>H</td><td>-2.01201600</td><td>-2.26615600</td><td>0.88735800</td></tr>
<tr><td>O</td><td>-1.16658400</td><td>-0.69107900</td><td>2.44983500</td><td></td></tr>
</table>

H -6.89123200 2.16417600 -1.51373600<br>O -1.50842700 0.66190000 2.42370200<br>H -2.47714200 0.66801900 2.41681900	
5FQA W RAF C8 dianion TS	
Cartesian Coordinates	Frequency and Energy
C -3.32728300 -0.42771900 -0.07400000<br>C -3.92784000 0.77989100 -0.54644000<br>C -5.27629400 0.98138200 -0.46121200<br>C -6.15810200 -0.02271800 0.12484700<br>C -5.52372600 -1.22476100 0.59422200<br>C -4.17580500 -1.41928100 0.50023500<br>C -1.92507000 -0.57525000 -0.16186700<br>C -1.21681400 -1.68736100 0.24526500<br>C 0.24177200 -1.78613600 0.09833000<br>O 0.83654700 -0.59875600 -0.08446000<br>O 0.84886000 -2.83906700 0.17280000<br>C 2.26872200 -0.54881300 -0.22408200<br>C 2.76035800 0.67535800 0.53223100<br>C 4.27659200 0.81536900 0.37236000<br>C 4.64866300 0.90007700 -1.11479400<br>C 4.12171300 -0.29471000 -1.90355300<br>C 2.61095700 -0.45608800 -1.70670300<br>O 2.12618800 -1.58241700 -2.42013400<br>O 4.90152700 -0.33496900 0.94953900<br>O -7.39936600 0.17015700 0.21343600<br>O -5.92893500 2.09520600 -0.88850400<br>O 4.78768500 -1.51271600 -1.55951700<br>C 4.78326800 2.08096200 1.10368700<br>O 4.29639500 3.17958100 0.75745100<br>O 5.66187000 1.89390900 1.98273100<br>H -3.27764400 1.53273900 -0.97582300<br>H -6.16792600 -1.97573900 1.03841800<br>H -3.74467400 -2.33802900 0.88198800<br>H -1.36631000 0.28594300 -0.51476900<br>H 2.69275800 -1.46033500 0.20057500<br>H 2.27266800 1.56849200 0.13334900<br>H 2.50728300 0.58145200 1.59134300<br>H 4.21629700 1.81194200 -1.53356000<br>H 5.73582500 0.95869900 -1.21818800<br>H 4.30478500 -0.14384600 -2.96963300<br>H 2.10579900 0.41875600 -2.12692600<br>H 2.65384000 -2.34367000 -2.14238400<br>H 5.55450700 0.02240300 1.57562300<br>H 4.98209700 -1.48201800 -0.61028400<br>H -1.70005300 -2.64149100 0.40434600<br>O -0.92880700 -1.50206500 2.33042000<br>O -1.42280800 -0.23751800 2.67293000<br>H -2.38571000 -0.33914700 2.64912800<br>C -5.13949900 3.12696300 -1.46903700	Zero-point Energy Correction = 0.357509 Hartree<br>Thermal Correction to Energy = 0.38454 Hartree<br>Thermal Correction to Enthalpy = 0.385485 Hartree<br>Thermal Correction to Free Energy = 0.29705 Hartree<br>EE + Zero-point Energy = -1486.4316 Hartree<br>EE + Thermal Energy Correction = -1486.4046 Hartree<br>EE + Thermal Enthalpy Correction = -1486.4036 Hartree<br>EE + Thermal Free Energy Correction = -1486.4921 Hartree

H	-4.41222700	3.51011300	-0.74850700	
H	-4.62063700	2.76254700	-2.35953300	
H	-5.83362000	3.91744800	-1.74561000	
TS 5CQA 12OH PE FHT				
Cartesian Coordinates				Frequency and Energy
C	-3.42920400	-1.99580800	-0.31791900	Zero-point Energy Correction = 0.351946 Hartree
C	-2.46422800	-0.97638000	-0.14450600	Thermal Correction to Energy = 0.37776 Hartree
C	-2.85176900	0.21481700	0.45611500	Thermal Correction to Enthalpy = 0.378705 Hartree
C	-4.17198300	0.39375000	0.89074100	Thermal Correction to Free Energy = 0.292615 Hartree
C	-5.11923300	-0.65740100	0.71749800	
C	-4.73498500	-1.84980000	0.10911900	EE + Zero-point Energy = -1448.0433 Hartree
O	-4.59504600	1.50634500	1.46585000	EE + Thermal Energy Correction = -1448.0175 Hartree
O	-6.36977500	-0.48552200	1.13970200	EE + Thermal Enthalpy Correction = -1448.0166 Hartree
C	-1.09995400	-1.22616900	-0.60969900	
C	-0.02385500	-0.46622000	-0.37102900	EE + Thermal Free Energy Correction = -1448.1027 Hartree
C	1.27994000	-0.86500700	-0.92564600	
O	1.48505700	-1.85987500	-1.59153000	
H	-3.12791300	-2.92110600	-0.79717600	
H	-2.15895700	1.03671600	0.60059800	
H	-5.46648600	-2.63713700	-0.02499500	
H	-4.78658900	2.25632000	0.66806400	
H	-6.43487200	0.39448200	1.54470300	
H	-0.95715400	-2.12989400	-1.19817800	
H	-0.04678100	0.43859600	0.22377700	
O	-5.00523000	2.79385800	-0.46967500	
O	-5.11097200	1.71729600	-1.28781400	
H	-4.22264900	1.59298300	-1.66623700	
O	2.23537100	0.01737000	-0.61366400	
C	3.61700900	-0.30044300	-0.91202900	
C	4.42585500	0.49951600	0.09961400	
C	3.96556600	0.09224600	-2.34673400	
H	3.76089400	-1.37495900	-0.76783200	
C	5.93476800	0.31027200	-0.13062500	
H	4.16483200	1.55634900	0.00551600	
H	4.16522900	0.16796900	1.10741000	
C	5.47591100	0.00365500	-2.59648000	
O	3.36380100	-0.74792400	-3.31928200	
H	3.64678400	1.13140300	-2.50118100	
C	6.29380100	0.75673400	-1.55928000	
O	6.31229600	-1.02301100	0.11646900	
C	6.70708300	1.17446300	0.86582700	
O	5.88149200	-1.36448200	-2.57317500	
H	5.67188900	0.42108800	-3.58994000	
H	2.67061400	-1.27971000	-2.90241400	
H	6.09717700	1.82583900	-1.66445900	
H	7.35806000	0.58585900	-1.74232600	
H	6.25516500	-1.50338200	-0.72152400	
O	6.33897300	2.46642600	0.78526600	
O	7.54374400	0.78465400	1.62788700	

H 5.25408200 -1.85027200 -3.12500500<br>H 6.86437000 2.96748900 1.42738800	
TS 5CQA 13OH PE FHT	
Cartesian Coordinates	Frequency and Energy
C 3.10178200 0.18209500 -0.40238300<br>C 4.16914400 0.98186000 0.00571400<br>C 5.47462500 0.60127800 -0.27719800<br>C 5.72668900 -0.60108500 -0.98848100<br>C 4.63873400 -1.40014500 -1.39818400<br>C 3.35050100 -1.01807500 -1.10996700<br>C 1.74942900 0.63715200 -0.08221300<br>C 0.60560800 0.02164600 -0.40680400<br>C -0.67872000 0.63170900 -0.01817900<br>O -1.71344100 -0.07855000 -0.47695000<br>O -0.80216200 1.65271500 0.62640200<br>C -3.05670300 0.26055100 -0.05358000<br>C -3.83986300 -1.03622400 -0.20984800<br>C -5.32385100 -0.83494600 0.10241200<br>C -5.90979600 0.24654000 -0.81457500<br>C -5.14086500 1.54952400 -0.68041800<br>C -3.63463800 1.38471800 -0.91429200<br>O -3.07057100 2.65413600 -0.62547900<br>O -5.51422800 -0.52159700 1.47816500<br>O 6.98479100 -0.90653600 -1.25408000<br>O 6.49983300 1.36482400 0.11224300<br>O -5.36181100 2.05011700 0.63953600<br>C -6.06825100 -2.15237600 -0.15856700<br>O -6.80907900 -2.59026600 0.85454700<br>O -6.00072400 -2.73975100 -1.20318900<br>H 3.99262200 1.90456500 0.54692500<br>H 4.85067400 -2.31050000 -1.94823600<br>H 2.52535700 -1.63970300 -1.43441100<br>H 1.68119400 1.57225100 0.46877900<br>H 0.55735500 -0.90913000 -0.95732500<br>H -3.01769400 0.57270300 0.99417400<br>H -3.74071800 -1.39546000 -1.23730500<br>H -3.41995200 -1.79092000 0.45906700<br>H -5.85070100 -0.10420100 -1.84743100<br>H -6.96256200 0.41619400 -0.57245200<br>H -5.51403200 2.27609200 -1.40939400<br>H -3.46978700 1.12636700 -1.96850300<br>H -2.23469800 2.53732200 -0.15141100<br>H -5.51922100 0.44438500 1.57336000<br>H 7.31598000 -1.67669000 -0.54417500<br>H -4.75243500 2.79042400 0.76334200<br>O 7.38638600 -2.44140500 0.50290500<br>O 6.36030900 -1.98644700 1.26017700<br>H 5.60356100 -2.55978700 1.04386400<br>H 7.31995000 0.95151000 -0.19994300	Zero-point Energy Correction = 0.352706 Hartree<br>Thermal Correction to Energy = 0.378208 Hartree<br>Thermal Correction to Enthalpy = 0.379153 Hartree<br>Thermal Correction to Free Energy = 0.294597 Hartree<br>EE + Zero-point Energy = -1448.0494 Hartree<br>EE + Thermal Energy Correction = -1448.0239 Hartree<br>EE + Thermal Enthalpy Correction = -1448.0229 Hartree<br>EE + Thermal Free Energy Correction = -1448.1075 Hartree

H -6.69576100 -1.96192700 1.59013300	
TS 5CQA 12OH aninon W FHT	
Cartesian Coordinates	Frequency and Energy
C -3.44338700 -1.99949800 -0.28176600<br>C -2.45623300 -1.00908500 -0.06424200<br>C -2.83449100 0.19835200 0.50652500<br>C -4.16802200 0.42412400 0.86910600<br>C -5.14290100 -0.58955000 0.63615300<br>C -4.76625600 -1.80038500 0.05937200<br>O -4.56505800 1.55938700 1.43668300<br>O -6.42307600 -0.37537900 0.96313500<br>C -1.07871800 -1.30983900 -0.45651400<br>C -0.02209100 -0.49894500 -0.32470800<br>C 1.31075500 -0.94649400 -0.76355900<br>O 1.56787900 -2.02623000 -1.25726500<br>H -3.14540800 -2.94053900 -0.73115700<br>H -2.12356100 0.99481900 0.69425000<br>H -5.51713800 -2.56173600 -0.11200500<br>H -5.01230500 2.19264000 0.65688200<br>H -6.51201500 0.50117800 1.37136100<br>H -0.92059500 -2.29226000 -0.89539700<br>H -0.08325000 0.49606600 0.09832200<br>O -5.46121600 2.63043100 -0.48629700<br>O -5.28237400 1.54812800 -1.28416400<br>H -4.39729300 1.65249000 -1.67713600<br>O 2.22987000 0.00431400 -0.53786700<br>C 3.60211600 -0.25693100 -0.89712000<br>C 4.47504500 0.45356400 0.12271300<br>C 3.86422800 0.28288900 -2.29683300<br>H 3.77027900 -1.33495600 -0.87175100<br>C 5.95917700 0.23375300 -0.19621200<br>H 4.25292500 1.52375100 0.09900300<br>H 4.25854000 0.07287800 1.12399000<br>C 5.33170500 0.07021000 -2.68171900<br>O 2.98906700 -0.28735300 -3.26005600<br>H 3.66072800 1.35810000 -2.30070500<br>C 6.26542200 0.66750700 -1.63588900<br>O 6.27832700 -1.14272500 -0.01655100<br>C 6.81266500 1.07340300 0.79240300<br>O 5.61316000 -1.32072100 -2.84055900<br>H 5.50184800 0.57642300 -3.63745000<br>H 2.95392900 -1.24294200 -3.12090900<br>H 6.16495600 1.75420200 -1.68965300<br>H 7.29976200 0.41348700 -1.88242400<br>H 6.91057600 -1.14868200 0.72203700<br>O 6.67934500 2.31627900 0.76418200<br>O 7.57016000 0.42050500 1.55483500<br>H 5.17023800 -1.62468300 -3.64119000	Zero-point Energy Correction = 0.337551 Hartree<br>Thermal Correction to Energy = 0.363438 Hartree<br>Thermal Correction to Enthalpy = 0.364383 Hartree<br>Thermal Correction to Free Energy = 0.279071 Hartree<br>EE + Zero-point Energy = -1447.6225 Hartree<br>EE + Thermal Energy Correction = -1447.5966 Hartree<br>EE + Thermal Enthalpy Correction = -1447.5957 Hartree<br>EE + Thermal Free Energy Correction = -1447.681 Hartree
TS 5CQA 13OH anion W FHT	

Cartesian Coordinates	Frequency and Energy
C 3.05450200 0.07478400 -0.33730500<br>C 4.13923900 0.84203900 0.08698700<br>C 5.43764500 0.45711000 -0.22270900<br>C 5.66676900 -0.73090900 -0.96850000<br>C 4.56150300 -1.50625700 -1.37446500<br>C 3.28087900 -1.10937800 -1.07766200<br>C 1.71260700 0.53897700 0.01121700<br>C 0.56025900 -0.03287000 -0.35939700<br>C -0.72185100 0.55504700 0.07310200<br>O -1.75615600 -0.13733300 -0.42240000<br>O -0.84348900 1.53003600 0.78655700<br>C -3.09496700 0.27818000 -0.07745500<br>C -3.95164300 -0.97523700 -0.03348500<br>C -5.40203500 -0.62300200 0.32430900<br>C -5.95016800 0.43537200 -0.64162000<br>C -5.05285700 1.65548800 -0.80898000<br>C -3.61527000 1.24075900 -1.13601200<br>O -2.77362900 2.37490100 -1.29102900<br>O -5.45174000 -0.14061000 1.66335600<br>O 6.90421900 -1.08032800 -1.28980700<br>O 6.53092800 1.14966800 0.14553600<br>O -5.08832300 2.44831000 0.37779600<br>C -6.26093300 -1.91307600 0.23054900<br>O -6.36950100 -2.45974000 -0.88871000<br>O -6.77103500 -2.31184300 1.30853900<br>H 3.97717900 1.75069200 0.65817800<br>H 4.75851600 -2.41316700 -1.93391300<br>H 2.44520100 -1.71446200 -1.40594800<br>H 1.66849100 1.43560500 0.62458800<br>H 0.50384300 -0.92078100 -0.97652800<br>H -3.06574900 0.76665200 0.89846900<br>H -3.92611500 -1.45791800 -1.01421000<br>H -3.55256000 -1.67096700 0.70873100<br>H -6.05903000 -0.02839400 -1.62497900<br>H -6.94149700 0.75448500 -0.30945300<br>H -5.42406900 2.25506800 -1.64657300<br>H -3.61701100 0.72831600 -2.10320500<br>H -2.50341000 2.68359200 -0.41622300<br>H -5.95940700 -0.81699500 2.14323500<br>H 7.43936000 -1.32674300 -0.38026300<br>H -4.68047200 3.29886800 0.17766300<br>O 7.70049600 -1.73975700 0.85886200<br>O 6.49939000 -1.56131600 1.46452300<br>H 6.04420300 -2.41987000 1.40448200<br>H 6.27993700 1.93014700 0.65926300	Zero-point Energy Correction = 0.337554 Hartree<br>Thermal Correction to Energy = 0.363494 Hartree<br>Thermal Correction to Enthalpy = 0.364438 Hartree<br>Thermal Correction to Free Energy = 0.279146 Hartree<br>EE + Zero-point Energy = -1447.6214 Hartree<br>EE + Thermal Energy Correction = -1447.5955 Hartree<br>EE + Thermal Enthalpy Correction = -1447.5945 Hartree<br>EE + Thermal Free Energy Correction = -1447.6798 Hartree
TS 5CQA RAF C8 anion W	
Cartesian Coordinates	Frequency and Energy
C -3.53091600 -0.16615400 -0.05188100	Zero-point Energy Correction = 0.342051 Hartree

C -4.02946300 0.81884400 -0.92956300<br>C -5.38292100 1.06276700 -1.01851100<br>C -6.27848800 0.33310700 -0.22019600<br>C -5.79923400 -0.63808700 0.65553000<br>C -4.44201500 -0.88953200 0.74147400<br>C -2.10964600 -0.37116800 -0.00202400<br>C -1.46436200 -1.30110800 0.78671300<br>C -0.01101200 -1.54924700 0.64079600<br>O 0.64455500 -0.49523700 0.15542100<br>O 0.51696500 -2.59296700 0.96576700<br>C 2.07761400 -0.57713700 -0.00372900<br>C 2.63137300 0.81075800 0.26773200<br>C 4.15549800 0.82548200 0.09488000<br>C 4.54166400 0.29252900 -1.29107300<br>C 3.91123000 -1.04945200 -1.64480300<br>C 2.39511600 -1.01550800 -1.42649600<br>O 1.79239700 -2.26134900 -1.74696600<br>O 4.75235400 0.03020900 1.11411000<br>O -7.61292200 0.56304200 -0.29041900<br>O -5.95009100 1.99060300 -1.84685300<br>O 4.51072300 -2.08456100 -0.86448300<br>C 4.65781800 2.28672800 0.24777700<br>O 4.26472400 3.12727900 -0.59060600<br>O 5.42059500 2.50595000 1.22306300<br>H -3.34478700 1.39156100 -1.54764700<br>H -6.51125000 -1.18463700 1.26235300<br>H -4.08797900 -1.64488300 1.43200100<br>H -1.50291900 0.30284700 -0.59812500<br>H 2.46640500 -1.30297900 0.71269800<br>H 2.18038800 1.51670400 -0.43494400<br>H 2.37581100 1.11831800 1.28492300<br>H 4.20681500 1.01833900 -2.03605300<br>H 5.62976800 0.21502600 -1.36244600<br>H 4.09097200 -1.25599800 -2.70462500<br>H 1.96614400 -0.28540800 -2.11966200<br>H 1.96407500 -2.88506000 -1.02919800<br>H 5.25623200 0.66572200 1.65044500<br>H -7.79001800 1.26199400 -0.93651500<br>H 4.28169900 -2.93216900 -1.26256500<br>H -2.00005400 -2.13063900 1.23135600<br>O -1.26053900 -0.47422100 2.57488100<br>H -5.27062500 2.43820500 -2.36783000<br>O -0.85053800 0.83037100 2.47526200<br>H -1.67000300 1.34317900 2.39265700	Thermal Correction to Energy = 0.368028 Hartree<br>Thermal Correction to Enthalpy = 0.368972 Hartree<br>Thermal Correction to Free Energy = 0.283662 Hartree<br>EE + Zero-point Energy = -1447.618 Hartree<br>EE + Thermal Energy Correction = -1447.592 Hartree<br>EE + Thermal Enthalpy Correction = -1447.5911 Hartree<br>EE + Thermal Free Energy Correction = -1447.6764 Hartree
TS 5FQA RAF C8 anion W	
Cartesian Coordinates	Frequency and Energy
C -3.27647900 -0.32277800 -0.08427800<br>C -3.83593400 0.96756600 -0.23960600<br>C -5.20376200 1.14140700 -0.23519800	Zero-point Energy Correction = 0.370855 Hartree<br>Thermal Correction to Energy = 0.398049 Hartree<br>Thermal Correction to Enthalpy = 0.398993 Hartree

C	-6.05052600	0.02629900	-0.06956900	Thermal Correction to Free Energy = 0.310996 Hartree<br>EE + Zero-point Energy = -1486.877 Hartree<br>EE + Thermal Energy Correction = -1486.8498 Hartree<br>EE + Thermal Enthalpy Correction = -1486.8489 Hartree<br>EE + Thermal Free Energy Correction = -1486.9369 Hartree
C	-5.51235200	-1.24509100	0.08608100	
C	-4.13936800	-1.42289000	0.07960200	
C	-1.84639600	-0.44448500	-0.09165100	
C	-1.15573200	-1.62525000	0.08539000	
C	0.30700100	-1.70730900	-0.16342400	
O	0.92891600	-0.54132700	0.00557500	
O	0.86010400	-2.74550300	-0.45561700	
C	2.34498000	-0.46157900	-0.26332700	
C	2.95370700	0.47878500	0.76325600	
C	4.44916700	0.65138500	0.48649100	
C	4.66935400	1.17427800	-0.94063300	
C	4.02193200	0.27048500	-1.98566100	
C	2.53482800	0.06606600	-1.68110400	
O	1.93041200	-0.79090100	-2.63555800	
O	5.08585800	-0.62251500	0.62559500	
O	-7.39550400	0.18876900	-0.06090000	
O	-5.85791400	2.32564400	-0.37717700	
O	4.67667800	-0.99577000	-2.09311400	
C	5.08022200	1.63909700	1.49663700	
O	4.57512400	2.77962600	1.57956700	
O	6.06921900	1.20659800	2.14077300	
H	-3.17207500	1.81427600	-0.36269200	
H	-6.18815200	-2.08256400	0.21127200	
H	-3.74065700	-2.42201400	0.20250200	
H	-1.27709900	0.47476100	-0.18595600	
H	2.77240900	-1.46200400	-0.17372400	
H	2.46132400	1.45252600	0.69984500	
H	2.80673200	0.07537800	1.76808300	
H	4.22982400	2.17140100	-1.02266800	
H	5.74156500	1.25605300	-1.13945700	
H	4.09904400	0.73273900	-2.97215300	
H	2.02474600	1.03044900	-1.76392400	
H	2.45370500	-1.60361900	-2.66534200	
H	5.83199000	-0.46273400	1.22896600	
H	-7.60418900	1.12670800	-0.18426100	
H	4.97075400	-1.24860500	-1.20466500	
H	-1.65270700	-2.58126800	-0.02049700	
O	-0.94789000	-1.83186400	2.02673800	
O	-2.20739500	-1.85158900	2.57417500	
H	-2.54415400	-2.74506200	2.39934900	
C	-5.06072700	3.49818700	-0.53273000	
H	-4.42236800	3.64507200	0.34152300	
H	-4.44976400	3.42931200	-1.43577200	
H	-5.76128000	4.32480300	-0.62254100	
TS 5FQA 13OH FHT P				
Cartesian Coordinates				Frequency and Energy
C	3.09105500	0.05135700	-0.34752100	Zero-point Energy Correction = 0.381077 Hartree<br>Thermal Correction to Energy = 0.408055 Hartree
C	4.18018000	0.82955600	0.06390100	

C	5.47952700	0.39901300	-0.16326800	Thermal Correction to Enthalpy = 0.408999 Hartree
C	5.71526400	-0.83755600	-0.84219900	Thermal Correction to Free Energy = 0.320789 Hartree
C	4.60015800	-1.61598400	-1.22595400	
C	3.31864900	-1.18055100	-0.99622600	EE + Zero-point Energy = -1487.3047 Hartree
C	1.75168900	0.56482900	-0.07950900	EE + Thermal Energy Correction = -1487.2777 Hartree
C	0.59171900	-0.04438400	-0.36068600	EE + Thermal Enthalpy Correction = -1487.2767 Hartree
C	-0.67517200	0.62972700	-0.02878400	
O	-1.72966500	-0.09649100	-0.41201200	EE + Thermal Free Energy Correction = -1487.3649 Hartree
O	-0.77267100	1.71103800	0.51553400	
C	-3.05802300	0.30332400	0.00426400	
C	-3.86532700	-0.98767600	-0.01034400	
C	-5.33675400	-0.72920500	0.32072700	
C	-5.93118800	0.27788000	-0.67226300	
C	-5.13324700	1.57029300	-0.68217300	
C	-3.64027400	1.34748800	-0.94838900	
O	-3.03738100	2.62431400	-0.81325300	
O	-5.48264600	-0.29159700	1.66780100	
O	6.92451600	-1.21443600	-1.15669000	
O	6.58753600	1.06260900	0.22373500	
O	-5.29863900	2.19431200	0.59267500	
C	-6.11184900	-2.04903800	0.19638100	
O	-6.81896600	-2.39118500	1.26892400	
O	-6.09510000	-2.71872700	-0.79979000	
H	3.99067300	1.77319100	0.55978800	
H	4.79765000	-2.55498900	-1.72923400	
H	2.48228600	-1.78821800	-1.31896100	
H	1.70201000	1.54132500	0.39698800	
H	0.51945200	-1.01887600	-0.82676000	
H	-2.99044800	0.71418600	1.01606000	
H	-3.80009600	-1.44250600	-1.00193500	
H	-3.44031100	-1.68524100	0.71488300	
H	-5.91224900	-0.16536700	-1.67043400	
H	-6.97203900	0.49133300	-0.41376800	
H	-5.51559300	2.23607700	-1.46262800	
H	-3.51907400	0.97747300	-1.97468000	
H	-2.19755200	2.53735600	-0.33962900	
H	-5.46911000	0.67868900	1.67668700	
H	7.58857100	-1.29149700	-0.25262800	
H	-4.67332100	2.93081800	0.62772600	
O	8.07687900	-1.54309300	0.86817800	
O	7.07105400	-1.26616600	1.74087900	
H	7.01024800	-0.29469500	1.72964400	
C	6.42976500	2.37168000	0.77517500	
H	5.92022300	3.02599900	0.06425900	
H	5.87575500	2.33377000	1.71684300	
H	7.43766900	2.73784000	0.95614300	
H	-6.67111500	-1.70579100	1.94500000	
TS 5FQA 13OH FHT P				
Cartesian Coordinates				Frequency and Energy

C	3.07828200	0.11251800	-0.31392500	Zero-point Energy Correction = 0.365891 Hartree
C	4.16262600	0.86547700	0.14481700	Thermal Correction to Energy = 0.393284 Hartree
C	5.46243300	0.48398000	-0.17155700	Thermal Correction to Enthalpy = 0.394228 Hartree
C	5.68686700	-0.68957100	-0.94997100	Thermal Correction to Free Energy = 0.305769 Hartree
C	4.58300900	-1.44276500	-1.38993500	
C	3.30211100	-1.04582800	-1.09080900	EE + Zero-point Energy = -1486.8769 Hartree
C	1.73579500	0.56370900	0.04995100	EE + Thermal Energy Correction = -1486.8496 Hartree
C	0.58440700	0.00093000	-0.33749000	EE + Thermal Enthalpy Correction = -1486.8486 Hartree
C	-0.70009500	0.56082200	0.12345400	
O	-1.72941900	-0.12573100	-0.39035700	EE + Thermal Free Energy Correction = -1486.9371 Hartree
O	-0.82807400	1.50738100	0.87316100	
C	-3.07136500	0.25203700	-0.01602600	
C	-3.90292400	-1.01912100	-0.00882900	
C	-5.35652500	-0.70858800	0.37148900	
C	-5.93314200	0.36955600	-0.55513300	
C	-5.06415200	1.61471200	-0.68214000	
C	-3.62024400	1.24321500	-1.03314700	
O	-2.80309400	2.39948900	-1.14991200	
O	-5.40418200	-0.27137800	1.72585700	
O	6.92323500	-1.04250500	-1.26748400	
O	6.57397600	1.12634200	0.21809700	
O	-5.10896100	2.36264100	0.53338900	
C	-6.19335200	-2.01023400	0.24204300	
O	-6.29005900	-2.53012800	-0.89103100	
O	-6.70095000	-2.44352100	1.30780500	
H	3.97612600	1.74951100	0.74134800	
H	4.77923500	-2.33415300	-1.97389100	
H	2.46607900	-1.63622600	-1.44387400	
H	1.69003600	1.43811200	0.69462000	
H	0.52978100	-0.86478600	-0.98542200	
H	-3.04053300	0.70466500	0.97707800	
H	-3.87639800	-1.46691500	-1.00585900	
H	-3.48394300	-1.73165800	0.70596000	
H	-6.03518100	-0.06183100	-1.55382900	
H	-6.93015500	0.65397700	-0.20872200	
H	-5.45407000	2.23653900	-1.49457900	
H	-3.61818400	0.76828800	-2.01938900	
H	-2.53516600	2.68160500	-0.26551900	
H	-5.90607400	-0.96662500	2.18447900	
H	7.46632600	-1.26915500	-0.36133400	
H	-4.72108900	3.22889900	0.36226400	
O	7.77441900	-1.66125000	0.88017200	
O	6.57530300	-1.60675500	1.51312800	
H	6.54299300	-0.72948000	1.93653300	
C	6.42495700	2.23936900	1.10395100	
H	5.87012200	3.04285100	0.61591100	
H	5.91560400	1.92847000	2.01890000	
H	7.43480600	2.56992900	1.33227200	
